# Smartphone Monitoring of Participants’ Engagement With Home Practice During Mindfulness-Based Stress Reduction: Observational Study

**DOI:** 10.2196/14467

**Published:** 2020-01-14

**Authors:** Christine E Parsons, Maria A Madsen, Kasper Løvborg Jensen, Simon Kæseler, Lone Overby Fjorback, Jacob Piet, Andreas Roepstorff, Conor Linehan

**Affiliations:** 1 Aarhus University Interacting Minds Center Aarhus Denmark; 2 Aarhus University School of Engineering Aarhus Denmark; 3 University College Cork School of Applied Psychology Cork Ireland

**Keywords:** mindfulness, adherence, smartphone monitoring, meditation practice, habit formation

## Abstract

**Background:**

Standardized mindfulness training courses involve significant at-home assignments of meditation practice. Participants’ self-reported completion of these assignments has been correlated with treatment outcomes, but self-reported data are often incomplete and potentially biased. In addition, mindfulness teachers typically suggest that participants set aside a regular practice time, preferably in the morning, but the extent to which participants do this has not been empirically examined.

**Objective:**

This study aimed to analyze patterns of participant engagement with home practice in a mindfulness-based stress reduction course.

**Methods:**

We used a novel smartphone app to provide 25 participants with access to their daily practice assignments during the 8-week course. We analyzed data collected through our smartphone app to determine usage and listening patterns and performed analyses of the regularity and frequency of participant behavior.

**Results:**

We found that participants listened to a median of 3 of the 6 practice sessions per week, and they did not typically set aside a regular daily practice time. Across weekdays, participants practiced most frequently in the morning, but there was considerable variation in participants’ practice start times. On weekends, the peak practice time was in the evening.

**Conclusions:**

We suggest that it is feasible to integrate a smartphone-monitoring approach into existing mindfulness interventions. High-frequency smartphone monitoring can provide insights into how and when participants complete their homework, information that is important in supporting treatment engagement.

## Introduction

### “Home Practice” in Mindfulness Training Programs

Mindfulness training programs have become extraordinarily popular in the last two decades [1]. The most rigorously evaluated of these programs are mindfulness-based stress reduction (MBSR) and mindfulness-based cognitive therapy (MBCT). MBSR and, its derivative, MBCT are manualized interventions. They follow a standardized syllabus, involving 20 to 26 hours of formal mindfulness training during 8 weekly group classes (1.5-2.5 hours/class), 1 all-day (6 hour) class, and home practice of mindfulness (about 45 min per day, 6 days per week). Home practices include the body scan (typically a lying down meditation, focused on sensations in the body), yoga, and sitting meditations supported by audio guides. The time commitment required from participants is substantial, but there is now a growing evidence base for these 2 interventions in improving mental health outcomes [2,3].

Home practice, one of the core components of MBSR and MBCT, is thought to be critical for learning but can also be challenging for participants to complete [4]. A recent meta-analysis of 48 studies found that participants report completing around 30 min of home practice a day on average, somewhat less than the recommended 45 min [5]. The extent to which participants report completing this practice was also correlated with treatment outcome (but the precise temporal precedence of practice, and related changes in outcome, has not been established). Although the average practice time was 30 min, there was also considerable variability across participants: some reported doing even more than the assigned amounts and other participants completed relatively little (eg, in one study, participants completed only one-fourth of assigned practices, see also a systematic review by Lloyd et al [6]). Given that these courses are used to treat mental health difficulties, and that practice completion is plausibly related to outcomes, a better understanding of participant engagement with practice is required.

### Self-Reports, Missing Data, and the Timing of Practice

Two recent systematic reviews on home practice in MBCT and MBSR reported that nearly all studies to date have relied on self-report, retrospective, paper-based measures to monitor formal home practice. The problems with retrospective self-report are numerous and include memory lapses, socially desirable responding, and inaccurate recall, as well as loss of paper diaries [7]. These problems may be compounded where participants face mental health difficulties, which is often the case in mindfulness-based interventions. The few instances where technology-based recording methods have been used still required participants’ manual input. For instance, one study used an electronic device (logger) to track the length of their home practice [8]. Another study asked participants to log home practice via a web-based portal [9].

Several recent studies have used technology-based methods, such as smartphone app usage data, or Web portals to record participants’ practice time (a measure of the use of audio guides for practice) during mindfulness training. However, these studies have delivered their own mindfulness training courses rather than using the 2 standardized formats, MBSR or MBCT. Often, these training courses do not include a face-to-face group and have no human teacher, relying instead on automated and self-guided stand-alone programs that differ substantially from standardized formats. For instance, one study using a mindfulness app with patients with cancer assigned just 15 min of home practice to participants, substantially less than the 45 min assigned in MBCT or MBSR [10]. Just over half of the patients continued to use the mindfulness app consistently until week 10, completing a median number of exercises of 4 at week 1, to a median of 2 at week 10. Another study used an iPad app to record listening time during a 6-week mindfulness course, again with reduced home practice requirements [11]. Participants listened to around 23 min per day according to the app, with a drop to 16 min per day after the end of the 6-week course.

There is a clear gap in the implementation of automated recording methods for the courses for which the most clinical evidence has accrued, MBSR and MBCT. It is important that we understand participants’ practice behaviors during these specific courses, which are now widely delivered in many countries. Indeed, one recent article argued that mobile technology will be crucial in solving some of the major methodological challenges in mindfulness research [12]. These challenges include how to measure participants’ engagement with mindfulness practice rigorously, over the long-term, and with larger samples.

Although there are proponents of mobile technology for delivering mindfulness training, there has also been considerable concern that the technology itself may be disruptive to attentional capacities (for review, see [13]). For example, high-frequency digital media use has been associated with the emergence of new attention-deficit symptoms in a large longitudinal study [14]. As mindfulness training targets attentional capacities, there may be reasonable concerns as to the acceptability of a smartphone app, when smartphones are frequently viewed as a source of attention disruption [15]. Furthermore, nearly one-fifth of Americans (N=3511, 18%) report that technology use is a *very* or *somewhat* significant source of stress [16]. For individuals selecting to attend an MBSR course, prescribing technology use as part of the program may be incompatible with their reasons for participating (stress reduction).

Another key issue in MBSR and MBCT relates to the practical guidance given to course participants around the timing of their practice. It is often recommended that students dedicate *a specific time and particular place to practice* and that students might *wake up earlier and devote that time to practice* [17]. Such recommendations are consistent with numerous studies of health behavior change and theoretical models of habit formation (for review, see [18]), which emphasize the importance of repeating behaviors in consistent contexts. However, existing studies have not recorded or assessed when and how regularly MBSR and MBCT course participants practice. This information is of fundamental importance to both teachers and students in how they approach the assigned home practices, which can be challenging to complete.

To address this, we developed a smartphone app to provide participants with a convenient means to access their home practice guides during an MBSR course, while simultaneously recording their listening times. Smartphone-based tracking provides a means to better understand how and when people carried out their assigned home practice. By removing the requirement for participants to fill in paper diaries, we aimed to obtain more detailed measures of practice completion, with reduced demands on participants. In this exploratory study, we aimed to examine participant practice behavior during an 8-week MBSR program. We used a custom-developed app [19] that was designed to provide a simple user experience that did not conflict with any of the principles underpinning MBSR (eg, evaluating practice time as good or bad). We aimed to assess the number of practice sessions participants completed, the time of day participants practiced at, and the consistency of practice time.

## Methods

### Participants

We recruited participants from an MBSR course scheduled to run at the Danish Center for Mindfulness. Participants were invited to a face-to-face information session before the start of the course to decide if they would take part. Participants self-selected to attend this course and paid a fee (€467) and did not receive any compensation for taking part in this study. A total of 30 participants were initially registered on the course (women=21 and men=9).

All participants who consented to participate were provided with a smartphone (Motorola G5). The phones were preloaded with the custom-built Android app that contained the class teacher’s own mindfulness practice guides. Participants were asked to use this phone to access their guides and not as a replacement personal phone. Qualtrics online software was used to collect participants’ demographic and health-related information before the start of the MBSR course, midway through the course, and at the end of the course.

Participants were informed that their practice time data would not be shared with the teacher and would only be stored anonymously for research purposes. All participants provided written informed consent to take part. The study was conducted in accordance with the local Danish legal guidelines. There was no requirement for a formal ethical committee review because participants’ treatment was not affected by participation in the study. Participants were already signed up to participate in the MBSR course before consenting to take part in this study. The study was registered with the Danish Data Protection Agency (AU-2016-051-000001).

Datasets from 25 participants were collected (4 women and 1 man not included from the original course). One participant changed to a different course time, 3 deleted the app before returning the smartphone to the research team, and 1 accessed their guides via alternate means (downloading MP3 files). All participants were Danish speakers. In all, 2 reported being on sick leave from work during the MBSR course. Most of the participants reported high levels of education (Table 1), and the average age was 49.3 (SD 11.5) years.

**Table 1:** Participant demographic information (N=25).

Demographic variable		Value, n (%)
**Gender**		
	Male	9 (36)
	Female	16 (64)
**Living situation**		
	Alone	1 (4)
	Living with partner	21 (84)
	With children under 18 years	11 (44)
**Education**		
	Apprentice or vocational courses	3 (12)
	Medium long higher education (3-4 years at university level; eg, Bachelors)	8 (32)
	Further higher education (>4 years at university level; eg, Masters)	12 (48)
	Other education	2 (8)
**Occupation**		
	Employed	21 (84)
	Unemployed	1 (4)
	Retired	2 (8)
	Other	1 (4)

### Mindfulness-Based Stress Reduction Class

A teacher who had a doctoral degree in psychology and was certified by the Center for Mindfulness in Medicine, Health Care, and Society, United States, taught the face-to-face MBSR class. The MBSR class was delivered as recommended in 2.5-hour weekly group sessions over 8 weeks with a 7-hour silent retreat day and 45 min of formal daily homework 6 days per week [20]. For classes 1 and 2, the formal home practice is called the body scan that focuses on nonjudgemental awareness to sensations in the body. From classes 3 and 4, participants are asked to alternate between the body scan and yoga practice, with yoga emphasizing mindful movement, through a series of gentle stretches. From class 5, participants are assigned sitting mediation practice, focusing on awareness of the breath, and yoga practice. From class 6, participants are asked to alternate the body scan practice with yoga practice. From class 7, participants are asked to practice on their own, without any specific recording, although participants can use the audio guides if they wish. The final class (class 8) emphasizes *making the practice your own* and using the recordings if desired. Participants on average attended 7.1 of the 8 classes (SD 1).

### Smartphone App Design

We designed an Android smartphone app to present participants with home practice guides (body scan, seated meditation, and yoga) and to record the amount of home practice sessions that participants completed. The app was designed via a user-centered process, using focus group discussions with both mindfulness teachers and former students. The focus groups and the subsequent analysis followed and an experience-centered design approach, which was designed to *invite participants to creatively express something about themselves, their values, their relationships, and the ways they make sense of experience* [21]. The design process is reported in detail elsewhere [19]. In summary, the app was designed to include the teachers’ own specific practice guides, rather than guides from another teacher. The app also included features such as reminders to practice, a way of recording students’ motivation for participating in the course, and a diary.

The basic features of the finalized app were similar to those present in the Oxford mindfulness-based cognitive therapy app (Oxford MBCT). Specifically, students could select a guide, play and pause, and dim the screen. Participants could access visualizations of practice times (in minutes) from previous practice sessions using simple bar charts. These visualizations were designed to ensure that they did not provide any evaluation of the *correctness* of participants’ practice behavior. There was no comparison of participant behavior against the assigned practice amounts, simply a graphical illustration of the participant’s own behavior. The app also had a simple diary function, where participants could note anything important that occurred during their practice.

**Figure 1:**
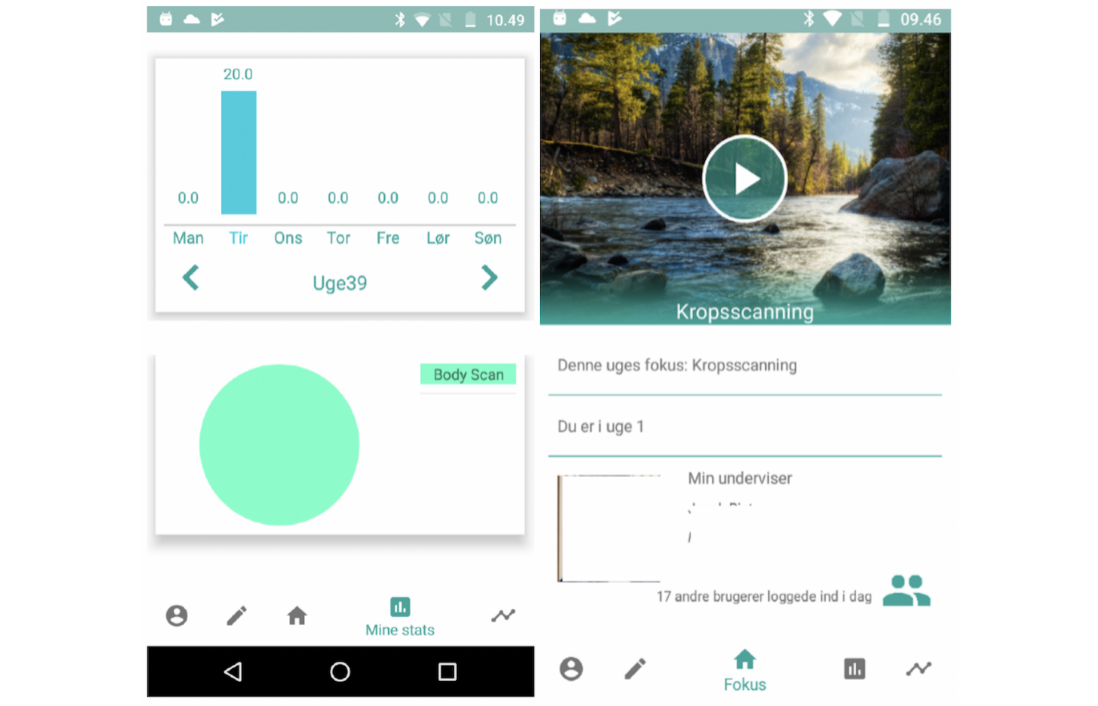
Screenshots from the app interface, illustrating the practice time visualizations (left) and the main screen with access to practice guides (right).

There were several features unique to our app. Although other apps have used mindfulness guides from well-known teachers (as in the Oxford MBCT app), we used guides prepared by the teacher who participants met in weekly face-to-face sessions. We presented these audio guides, along with a picture of the teacher. We also included a feature to allow participants to see how many others from their course logged into the app that day. The overall aim was to create a simple interface for participants that required minimal explanation or support from the research team. We wanted participants to be able to access their guides in a convenient manner and designed the app such that all other features were optional (such as, looking at their practice graphs, see Figure 1, or using the diary).

### Statistical Analysis

The metrics of interest were the frequency with which participants accessed the practice guides, the length of time spent listening to each of those guides, and the time of day at which those sessions were accessed. We analyzed participants’ data from 9 consecutive weeks, rather than the typical 7 from the MBSR course. This was because there were 2 national holidays that fell during the standard 8-week course (holidays occurred on class 4 and class 6). We focused our analyses on our first 8 weeks of data, which represented the portion of the course where participants were assigned specific practices. In the last week, the participants were encouraged to practice without an audio guide, as per the MBSR course manual.

Analyzing the time participants spent listening to the practice guides, we calculated the sum of minutes logged for the full day. This was to account for instances where participants logged in and did a proportion of their practice and logged in again later. Furthermore, the app did not automatically stop the audio file playing when the participant closed it. We set the maximum practice time per day to 60 min because the home practice guides were 45 min long, and because 60 min was the upper limit of self-reported home practice found in a recent meta-analysis [5]. We also repeated our analyses with the maximum practice time per day set to a more conservative 45 min, and this did not change the pattern of results obtained.

We examined the regularity of participants’ practice time using a number of strategies. First, we examined within-participant patterns of regularity, using the standard deviation from each participant’s mean practice start time. Second, we calculated a stability index for participants’ practice start time and used this to examine changes across the 8-week course. This method was adapted from a well-established measure in sleep timing research, where the regularity of sleep patterns is important (the Sleep Timing Questionnaire [22]). We assigned each participant’s standard deviation in practice times (for each week) as a number on a 9-point scale, with higher numbers indicating greater irregularity, based on the following intervals: 1: 0 to 1 hours, 2: 1 to 2 hours, 3: 2 to 3 hours, 4: 3 to 4 hours, 5: 4 to 5 hours, 6: 5 to 6 hours, 7: 6 to 7 hours, 8: 7 to 8 hours , 9: >8 hours.

### Self-Report Measures

The Symptom Check List-5 (SCL-5), a short form of the Hopkins Symptom Check List-90, was used to measure symptoms of anxiety and depression [23]. The Perceived Stress Scale (PSS-10) was used to measure the degree to which an individual perceived life as unpredictable, uncontrollable, and overloading during the previous month [24]. Table 1 provides all available data from the participant demographic questionnaires.

## Results

Table 2 presents all available data from participants’ PSS scores. Most participants self-reported experiencing moderate (52.2%) to high (13%) levels of stress (using cutoffs reported, for instance, in [25]).

**Table 2:** Self-report questionnaires: Perceived Stress Scale and Symptom Check List scores at baseline, midintervention, and postintervention.

Questionnaire	Preintervention		Midintervention		Postintervention	
	n (%)	Mean (SD)	n (%)	Mean (SD)	n (%)	Mean (SD)
Perceived Stress Scale-10	22 (100)	17.5 (6.9)	21 (100)	15.1 (5.3)	12 (100)	13.9 (6.1)
Low perceived stress (0-13)	7 (31.8)	—^a^	8 (38.1)	—	7 (58.3)	—
Moderate perceived stress (14-26)	12 (54.5)	—	12 (54.5)	—	5 (41.7)	—
High perceived stress (27-40)	3 (13.6)	—	1 (4.5)	—	0 (0)	—
Symptom Check List-5	22 (100)	10.9 (3.3)	22 (100)	10.7 (3.3)	17 (100)	9.2 (2.3)

^a^Not applicable.

### App Usage: Practice Listening Time

**Figure 2:**
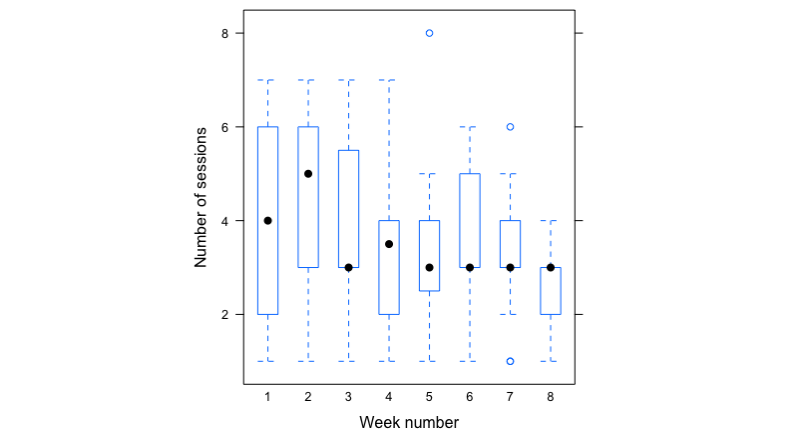
Box plot showing participants’ number of listening sessions recorded over the weeks of the course with assigned practice (week 1-8). The median listening time for each week is represented by black dots in each box, and the broken lines represent the minimum and maximum quantiles.

Participants completed practice sessions a median of 3 times per week from week 3 onward (see Figure 2). In the weeks with assigned home practices (weeks 1-8), participants (n=25) listened to a mean of 3.1 (SD 0.9) practice sessions weekly. In week 1, the mean number of sessions was 4.36 (SD 1.9). By week 3, participants’ mean number of sessions was around 3.48 (SD 2.5). In the final week, where participants did not have an assigned audio recording (the guidelines suggest doing practice without any guide, but participants could use an audio guide if they wished), 9 participants listened to recordings a total of 26 times. This suggests that course participants used the app throughout the course, even when they were not assigned a specific session that required app use.

On average, participants listened to 123.15 (SD 40.86) min of audio recordings per week across weeks 1 to 8. In the first week of the program, the average listening time was 187.05 min. A simple linear regression was used to examine trends in listening time over the 8 weeks with assigned practice. As the course progressed, participants spent significantly less time listening to the recordings (*F*_1314_=83.5; R^2^=0.06; beta=–0.35; *P*<.001). The most frequently accessed recording was the *body scan*. In total, participants played this for 443 sessions out of a total number of 793 sessions. Next most frequently accessed was the *yoga* recording that was played 183 times, and the *sitting meditation* was played 167 times.

### When Did Participants Practice?

**Figure 3:**
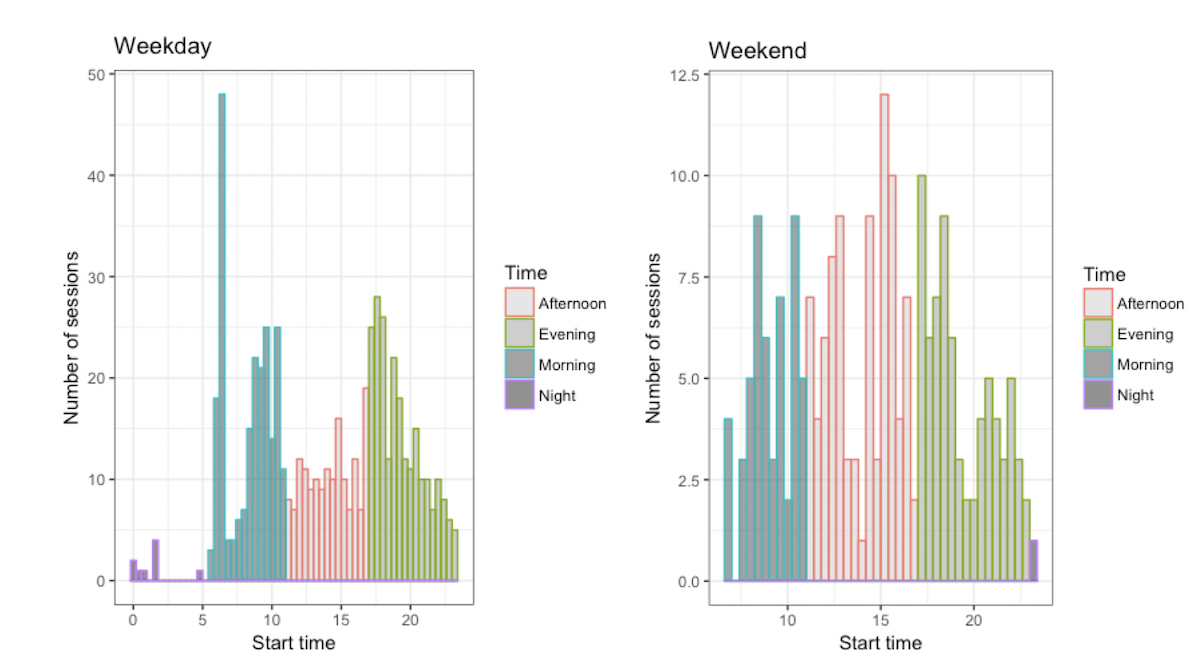
Histogram showing the start times and number of sessions completed in the morning, afternoon, and evenings, across weekdays and weekends.

The most common start time for a practice session was in the morning (between midnight and midday), in which 34.4% of sessions occurred (see Figure 2), and in the evening, in which another third (35.14%) of the sessions were completed. The afternoon (between midday and 17.00) was the second least popular time, with 29.7% of the listening times occurring in this time window; 0.7% of the sessions were done during night time. There were 2 distinct peaks in practice times, around 08:30 am and 7:45 pm on weekdays. At weekends there was a peak at around 7:00 pm (see Figure 3).

#### Practice Time Regularity

**Figure 4:**
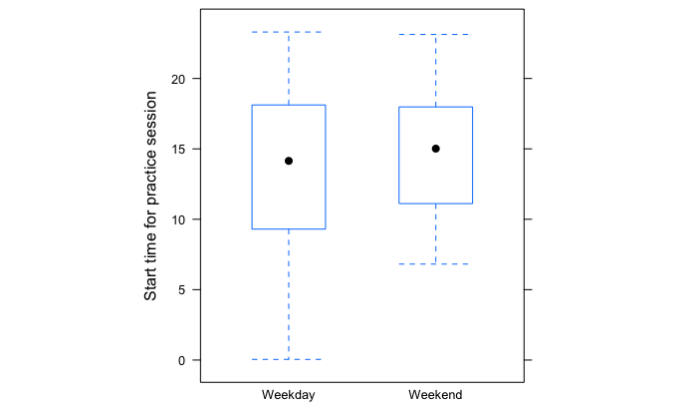
Box plot illustrating the variability in participants’ practice start times on weekdays and weekends. The whiskers represent the minimum and maximum quartile, showing the size of variability in practice start times, which was greater during weekdays. The median starting time is indicated by black dots.

Across the 25 participants, there was significant variability in the regularity of their listening times. Some practiced at similar times every day, whereas others did not. For instance, one participant had an overall standard deviation in practice time of 0.43 (corresponding to 25.8 min), and another had an overall practice time standard deviation of 8.17 (corresponding to approximately 8 hours and 10 min). The average standard deviation in listening time was SD 4.0 corresponding to 4 hours. Overall across all participants, there was more variability in start times across the weekdays (SD 5.21) compared with the weekends (SD 4.24, see Figure 4). Looking at within-participant variability, for weekdays, the average SD in start time was 3.78, and for weekends, it was 3.33.

The majority of participants (68%) had an overall stability index that corresponded to 3, 4, or 5 (between 2 and 5 hours of variation in practice start time). We also repeated this analysis looking specifically at weekdays, excluding weekends. The mean stability score for weekdays was 3.6 (SD 2.5), whereas for the full week it was 3.8 (SD 2.4), a difference that was statistically significant (*t*_125_=–1.96; *P*=.05). Participants therefore were slightly more regular in their weekday start times, as compared with the full week.

A linear regression showed no significant changes in participants’ stability indices over the full course (*F*_132_=0.09; R^2^=0.007; beta=0.02; *P*=.80). This indicates that participants did not adopt a more regular practice time as the course progressed. A linear regression model showed no significant relationship between the calculated stability index for practice time and the amount of listening time participants completed (*F*_21_=1.3; R^2^=0.01; beta=–87.36; *P*=.28) This suggests that, for the small sample included here, having a more regular practice time was not associated with greater practice listening time.

## Discussion

### Principal Findings

We used a smartphone app to monitor participants’ engagement with the home practice assignments within a standard format 8-week MBSR course. From this smartphone listening time data, we found wide variability in how much participants listened to their home practice guides, with a median of 3 sessions per week recorded, compared with the assigned 6 sessions. This number of sessions is similar to findings from previous studies using paper-based recording methods (eg, [5]). Notably, we were able to assess, for the first time, when and how regularly (ie, day-to-day variation) participants carried out their home assignments. Participants most frequently practiced in the morning, and in the early evening during the weekdays, and in the afternoon at weekends. Analysis of the time patterns suggested that participants were generally not setting aside a regular practice time, even when examining weekdays only.

### Number of Sessions and Timing of Practice

Our data indicate that participants used the app throughout the course, in a manner that is in line with previous paper-based monitoring studies. In fact, participants frequently used the app even during the week when they were told they could practice without it (week 7 in the MBSR program). This suggests that the smartphone-monitoring approach presented here is feasible to implement, and this allowed us to analyze key features of participants’ practice.

Participants’ median number of listening sessions was roughly comparable with findings of previous studies of standard format MBSR and MBCT courses (see systematic reviews by [5,6]). The wide variation in the number of practice sessions that people completed was also in line with general findings in the field. We also found that participants gradually listened to fewer guides as the course progressed. This is in line with other studies showing that participants report practicing the most in the early weeks of the course, with a gradual drop-off as the course proceeds [26]. The most commonly accessed guide was the body scan meditation that was logical, given that the body scan is the most frequently assigned activity in the program (ie, it is the only assigned practice for the first 2 weeks, and for other weeks, participants are asked to alternate between the body scan and other practices [27]).

Our key finding was that the most frequent practice time during weekdays was in the morning, where there was a clear peak in participants’ listening frequency. This suggests that many participants are setting some morning time to practice, in line with the seminal recommendations from Williams et al [17]. However, on weekends, this pattern shifted, and participants’ peak listening time was in the early evening. We performed analyses on the regularity of participants’ practice which indicated that, on average, participants did not stick to a consistent practice time from day to day. We found that participants were slightly more consistent in their start times across weekdays, compared with the full week, but there was still considerable weekday start time variability (approximately 3 hours).

This information on timing and regularity of practice is of clear importance to teachers and may be used to better support participants’ home practice. Research on habit formation suggests that consistency, choosing the same time and place for the target behavior, is of particular importance (eg, [28]). For mindfulness practice, teachers may helpfully address this, particularly for students who are having difficulty completing their home practice. Whether it is important that participants practice at the same time during the weekdays and weekends is an open question for future research. Students might be guided to reflect on their own routines, or lack thereof, to establish and maintain a mindfulness practice *habit*. Research on completion of other health-related behaviors suggests that weekends can be more problematic than weekdays. For instance, studies on medication-taking behavior show that weekend doses are more likely to be missed than weekday doses (eg, [29]), and having a medication routine (taking at the same time each day across the week) is associated with better adherence (eg, [30]).

We did not find evidence for an association practice regularity and overall practice time in our statistical analysis; this may have been because our sample size was small. The aim of this exploratory work was not to establish that such effects exist, rather to systematically measure home practice in a standard format MBSR course using a smartphone-based automated method. In particular, we were able to examine features of practice, timing, and regularity, which paper-based methodologies have not monitored. We believe this information is of interest to mindfulness teachers and students because we know that it is more difficult to repeatedly engage in a behavior if it is not part of a daily routine. This smartphone-monitoring approach, if scaled up, would allow us to test the importance of regular practice time for practice engagement and its association with outcomes.

### Limitations of the Smartphone-Monitoring Approach and This Study

There were several limitations to this approach. First, we assumed that participants used the smartphone app as their only means of listening to their mindfulness practice guides. This may not have been the case. There is an abundance of MP3 files available for free online (along with other popular apps, such as Headspace), and participants could have practiced with these, even though they are not strictly the assigned practices. It is also possible that participants did silent practices and not the assigned practices. Second, it is possible that participants pressed play on the guide within the smartphone app but did not actually listen to the guides. For instance, one participant in our user-centered design group suggested that she might *cheat* by pressing play on the recording and leave the phone while she engaged in other tasks [19]. We cannot exclude such a possibility.

Third, we had poor completion rates for the final follow-up mental health questionnaires, and we did not obtain a measure of state (eg, the Toronto Mindfulness Scale [31] or trait mindfulness (eg, the Five-Facet Mindfulness Questionnaire [32]) which would have been of interest. Poor questionnaire completion rates have been reported in similar self-paying participant samples [33]. Furthermore, 3 participants deleted the app before returning the smartphone to the research team. We took this as an indication that those individuals were opting out of participating in the study, in addition to the participant who chose to download MP3 files instead of using the app. These participants did not communicate a reason for deleting the app, and we did not have a procedure in place to ask about this.

It may be that those participants were uncomfortable sharing data on their practice behavior with the research team. Opting out of sharing data may be because of nonadherence, as is often discussed in relation to missing data on medication adherence [34]. It may also be because some adults are less comfortable sharing data related to mental health issues, compared with other aspects of health [35]. Although we informed participants that their practice data would not be shared with the class teacher, we could also have added a data sharing option within the app. This could have served a dual function: allowing participants to share data with their teacher if they wished and reminding participants explicitly that they could choose not to do this. For a small subset of participants, smartphone recording of treatment engagement may be undesirable, and they may prefer to use more traditional means of accessing their practice guides.

Finally, we acknowledge that this is a small sample, and the self-selecting nature of the participant group limits the potential generalizability of the findings. Through their self-selection, we can assume that our participants here were motivated to practice mindfulness, perhaps more so than participants who do not seek out this specific course, as now sometimes occurs in health care settings. This is important because there is evidence that patient preference for treatment can impact treatment outcome [36]. Indeed, there is evidence that baseline differences between adults before training can predict engagement with practice [37]. This suggests that there may be characteristics that differentiate those attracted to mindfulness from the general population [1].

### Strengths of the Study

The majority of participants used the app regularly across the course, although there was wide variation in the actual number of listening sessions recorded. Overall, this suggests that smartphone-based access to mindfulness guides is feasible and acceptable to most participants. There are several advantages to this approach: it offers a convenient and universal means for participants to access their home practice guides. Participants otherwise use a variety of MP3 players and desktop computers to access these and typically do not record their home practice completion.

Furthermore, we suggest that this approach will reduce the burden to the teacher of having to manually collect 25 to 30 home practice diary sheets from class participants. There have been numerous calls for better monitoring of participants’ *at-home* treatment engagement [5,6]. In fact, although recording of home practice completion is common in research studies, it is not widely carried out in community settings. In fact, the Danish Centre for Mindfulness, where we conducted this study, did not use practice recording forms, despite being a research-oriented center, because of the additional administrative task in doing so.

Smartphone recordings also reduce the burden on participants of having to fill in additional paperwork. This is important because it is often the case that a self-selecting and self-paying population (which is typical for MBSR) show low participation rates when asked to fill in questionnaires on their experiences during the MBSR program [33].

Although mindfulness-based apps are incredibly popular, the actual use of app-based monitoring with clinical populations, and in standardized mindfulness-based interventions, is limited. Mindfulness apps have been explored predominantly in academic research in nonstandardized and often brief training formats [38,39]. We bridge this gap, using a smartphone app in a real-world context, to support adults in a community setting who had already signed up to attend an MBSR course. This type of population is of clear interest and arguably distinct from those who already engage with health-related technology (adults who are health conscious and who want to quantify progress; [40]).

### Avenues for Future Work

The issue of the practice *dosage* in mindfulness training is among the most important practical questions [12] yet has received little research attention. By *scaling* this app for use with larger samples of participants, and multiple teachers, it will be possible to address outstanding questions in the field. For example, is it more effective to practice in multiple, brief sessions in a given day, or in one longer session, as currently recommend? Is it more sustainable to practice in the morning, and can this be maintained beyond an 8-week program? and Should participants be advised to practice at similar times across weekdays and weekends? At present, we do not know the answers to these questions.

The app-based approach presented here offers a feasible route to longer-term assessment of participants’ mindfulness practice behavior. This is of interest, because the majority of studies report on participants’ practice only during the 8 weeks of the MBSR or MBCT program [6], and the limited available evidence of within-participant changes suggests that higher practice engagement 2 to 6 months after training predicts lower levels of subsequent stress [41]. By relying on methods that require less concerted efforts from participants and researchers, it is likely that we can collect larger, more representative datasets on postintervention practice.

Although we focus on mindfulness-based interventions, there are numerous other evidence-based treatments where homework is a central component, such as Cognitive Behavioral Therapy (CBT). In CBT, patients are asked to record thoughts and emotions, plan activities, or track mood, and compliance with these homework exercises has been correlated with treatment outcomes (meta-analysis; [42]). It has been clearly argued elsewhere that well-designed apps might helpfully support patients in their CBT homework completion [43,44]. We further propose that the systematic recording of homework completion, its timing and consistency as we achieved here for MBSR, may also be of value. Studies examining the homework completion and CBT treatment outcome association have relied on paper-based self-reports for the most part [42], as for MBCT and MBSR. Smartphone monitoring offers a number of clear advantages in terms of convenience and the potential for automaticity.

We used our app to collect time-based metrics only, but smartphones provide a number of methods for measuring physiological signals relevant to mindfulness practice. Recent work has, for example, correlated heart rate measured with contact photoplethysmography (contact of fingertip to built-in camera) and electrocardiograms and reported reasonable accuracy [45]. Breathing rate has also been measured using noncontact video recordings of chest and abdominal motions, correlated with respiration belts measures, again with reasonable accuracy [46]. These physiological signal measurements might be integrated into smartphone monitoring of at-home mindfulness practice and would provide new ways to link laboratory-based experiments of practice with real-world measurements. Finally, our app did not directly assess participants’ user experience *in vivo* (eg, via an inbuilt feature*)*, and in-depth qualitative examinations of individuals’ experiences will be of clear importance for future development of the app.

### Conclusions

The 2 standardized mindfulness training courses with the most substantial body of clinical evidence, MBSR and MBCT, involve significant *at-home* assignments of formal mindfulness practice. Participants’ self-reported completion of formal home practice is associated with treatment outcomes, but self-reports are considered to be methodologically problematic. Furthermore, it is often recommended that course participants set aside a regular practice time, preferably in the morning. However, the extent to which participants do this has not been systematically examined. In this study, we used a novel smartphone app to provide participants with access to the daily at-home practice exercises during MBSR. We found that participants listened to a median of 3 of the 6 assigned practice sessions per week over the 8-week course. During weekdays, participants practiced most frequently in the morning, but there was considerably variation in participants’ practice start times. During the weekend, the peak practice time was in the evening. Overall the data suggested that participants did not set aside a regular daily practice time. We suggest that it is feasible to integrate a smartphone-monitoring approach into an existing, well-established mindfulness intervention, and this can provide valuable insights into participant behavior. This information may be helpful to both teachers, students, and researchers in establishing the most effective means to support treatment engagement.

## Abbreviations

CBT: cognitive behavioral therapy
MBCT: mindfulness-based cognitive therapy
MBSR: mindfulness-based stress reduction
PSS: Perceived Stress Scale
SCL: Symptom Check List
